# A Mini-Review: Clinical Development and Potential of Aptamers for Thrombotic Events Treatment and Monitoring

**DOI:** 10.3390/biomedicines7030055

**Published:** 2019-07-26

**Authors:** Alex T. Ponce, Ka Lok Hong

**Affiliations:** Department of Pharmaceutical Sciences, Nesbitt School of Pharmacy, Wilkes University, 84 W. South Street, Wilkes-Barre, PA 18766, USA

**Keywords:** thrombotic event, aptamer, DNA, RNA, SELEX, molecular recognition element (MRE), monitoring, treatment

## Abstract

The unique opportunity for aptamer uses in thrombotic events has sparked a considerable amount of research in the area. The short half-lives of unmodified aptamers in vivo remain one of the major challenges in therapeutic aptamers. Much of the incremental successful therapeutic aptamer stories were due to modifications in the aptamer bases. This mini-review briefly summarizes the successes and challenges in the clinical development of aptamers for thrombotic events, and highlights some of the most recent developments in using aptamers for anticoagulation monitoring.

## 1. Introduction

Antibodies and their variants, such as antigen-bind fragments (Fab) and single-chain variable fragments (scFv), have received considerable attention in the area of biomedicine. In the past decade, there has been a modest increase in the number of biological drugs approved by the US FDA [1]. Almost all of the approved biologics are antibodies and antibody–drug conjugates, with a few additional enzyme products [1]. A largely under-explored area of biologics is nucleic acid-based aptamer therapies. The purpose of this review is to provide a summary and perspective of the clinical success and failure of aptamer-based therapeutic agents in the treatment and monitoring of thrombotic events.

### 1.1. A Brief History of Aptamers

Aptamers are molecular recognition elements (MREs) that include peptides and nucleic acids (RNA and ssDNA). Nucleic acid aptamers have received high interest in their use as therapeutic agents, targeting agents, and biosensing elements. Aptamers, by definition, can bind to various user-defined targets with high affinity and specificity [2]. Tuerk and Gold first described the process of discovering oligonucleotide binding elements closed to three decades ago [3]. Tuerk conducted an in vitro selection experiment, which was first described as the Systematic Evolution of Ligands by Exponential Enrichment (SELEX), to further understand mutations in the gene 43 protein in bacteria. Tuerk decided to mutate a hairpin loop that contained eight nucleotides. The project produced two RNA binding elements (aptamers) that had an equal affinity to gene 43 [4]. Ellington and Szostak developed a similar process and named their products aptamers, which means “fitting part” [5]. After these initial experiments, the length of aptamers was expanded to include up to 50 nucleotides to gain further insight into the tertiary structure and folding of aptamers [4]. In 1992, NeXstar started using aptamer technology to develop therapeutic agents similar to antibodies. The first aptamer that underwent a clinical trial phase was NX1838. This compound was approved by the FDA in 2005, and is now on the market under the trade name Macugen^®^. Since then, many other researchers started experiments on the therapeutic, biosensing, and diagnostic applications of aptamers in many different areas of interest [6,7].

### 1.2. Aptamer Selection

**Figure 1:**
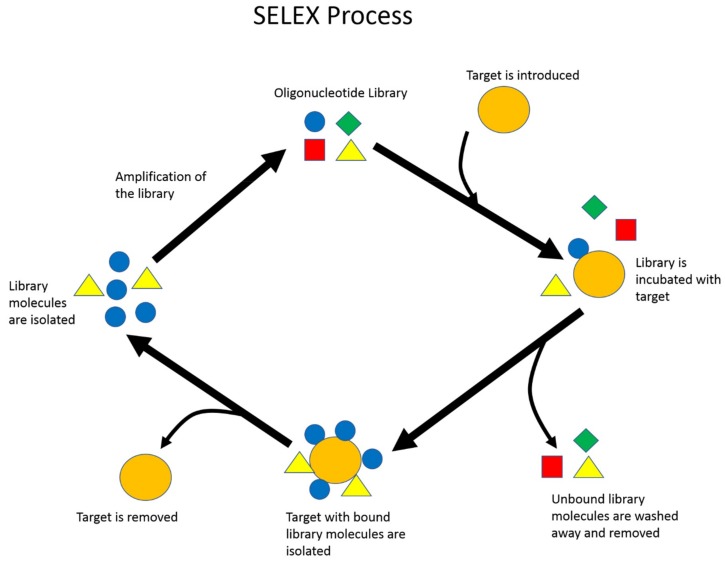
Illustration of the basic Systematic Evolution of Ligands by Exponential Enrichment (SELEX) process. Repeated cycles of target–library incubation, partitioning, and amplification are performed to enrich the library’s overall affinity toward the target of interest.

SELEX is the standard procedure to identify nucleic acid aptamers (Figure 1). First, a library composed of a random region surrounded by two constant regions for primer attachment is designed and chemically synthesized. The two constant regions allow the library to be amplified by a polymerase chain reaction (PCR) later on in the selection process. The diversity of this library is described to be 4^n^ diversity, where n represents the number of random nucleotide bases that compose the random region of the library. For single-stranded DNA (ssDNA) aptamer selection, the second step is to introduce the library to the desired target [7]. In comparison, RNA aptamer selection first requires PCR amplification to add a T7 promoter, and then in vitro transcription of the dsDNA library into an RNA library before incubation with the desired target. A selection target may be a small molecule, protein, enzyme, or a whole prokaryotic or eukaryotic cell. During library–target incubation, the two species are allowed to interact. Library sequences that do not bind to the target are washed and removed. The collected library sequences will be amplified with PCR, and the library strands are eventually retrieved from amplified double-stranded DNA (dsDNA). For RNA aptamer selection, the collected RNA molecules are reverse-transcribed back to cDNA before PCR amplification, and lastly transcribed back to RNA [8]. This population of enriched library molecules will be reintroduced to the target, and the process will be repeated, resulting in the enrichment of binding library molecules after each round. This process generally lasts somewhere between 10–15 cycles [9,10].

Throughout the years, many variations of the SELEX process have been created to improve the quality of the selection process. One method is to introduce negative targets or counter targets to remove aptamers with a higher affinity to undesired targets or have a high binding affinity for non-specific molecules. A SELEX process was developed that uses graphene oxide to interact with the random library, which does not require the immobilization of the target [11]. Other SELEX variants, such as capillary electrophoresis and microfluidics-based SELEX, are designed to increase the efficiency of liquid separation and partitioning. Detailed discussions of different SELEX methodologies have been extensively reviewed by several groups [7,12,13]. After the SELEX process is complete, library molecules are either cloned into competent cells for sequencing and analysis or more recently analyzed by high-throughput sequencing, due to decreasing cost. Several candidate sequences will often be selected and subjected to target-binding affinity and specificity characterization.

### 1.3. Clinical Advantages and Disadvantages of Aptamers

Nucleic acid aptamers have several unique properties that give them many advantages for clinical applications. The first quality that distinguished them from small molecule therapeutics is that they have high affinity and specificity for their targets due to the SELEX process [3]. Nucleic acid aptamers generally have a low potential for systemic toxicity due to their composition of natural molecules [3]. Additionally, aptamers can be synthesized chemically without the use of live systems. Chemically produced aptamers generally are non-immunogenic, which is a significant advantage over the immunoglobulin complexes currently on the market [3]. Aptamers are also more thermally stable than immunoglobulins, which allows them to be transported and stored more conveniently for practical purposes. Once an aptamer is selected, it is inexpensive to produce, which may give aptamers a cost advantage for therapeutic purposes in comparison to immunoglobulins [9,14]. The primary clinical disadvantage of an aptamer is the short half-life in the body [3]. It is mainly due to nucleases degradations and high renal clearance. Nucleases within the bloodstream quickly degrade aptamers, which significantly reduces concentrations in the body, and thus prevents them from reaching their target sites [15]. This phenomenon is particularly significant with RNA aptamers. Ni et al. have recently reviewed the topic of chemical modifications of nucleic acid aptamers [16]. Briefly, the in vivo resistance to nucleases can be enhanced by modifying the main areas of susceptibility, the 5’ and 3’ terminal ends, and the phosphodiesterase backbone [3]. Modification to the sugar ring of the nucleoside and creating mirror images of the nucleic acid aptamer (Spiegelmers) can also enhance resistance to nuclease degradation. Additions of long bulky chains of biocompatible molecules, such as cholesterol and polyethylene glycol, can increase aptamers resistance to renal clearance [16]. However, modifying these areas after selection may risk alterations in binding affinity or specificity [17].

More recently, both academia and the industry have reported the introduction of unnatural or modified nucleotides to the library during aptamer selection. SomaLogic Inc. reported using five-position modified dC and dU to select SOMamer for proprotein convertase subtilisin/kexin type 9 (PCSK9) protein. The Hirao group reported using two highly hydrophobic unnatural bases, 7-(2-thienyl)imidazo[4,5-b]pyridine (Ds) and 2-nitro-4-propynylpyrrole (Px), to expand the genetic alphabet of their library to select high-affinity DNA aptamers. They termed this new SELEX variant ExSELEX [18]. In both cases, the modification produced aptamers with high affinity and enhanced nuclease resistance.

Since the discovery, aptamers have been investigated for a wide array of therapeutic and biosensing applications. Many disease states have been researched [9]. The greatest success has been recognized in the treatment of age-related macular degeneration (ARMD), leading to the first approved aptamer drug, Macugen^®^ [5]. At the time of this review, ARMD is still the only FDA-approved aptamer for therapeutic applications. This may be because the clinical delivery of aptamer drugs to the localized area affected by ARMD has fewer pharmacokinetics challenges, i.e., adsorption, distribution, metabolism, and excretion. Nucleic acid aptamers that target various cancer cell surface proteins and biomarkers is a growing interest in both academia and the industry [19,20]. Aptamers have been shown to be used in conjunction with nanomaterials, such as carbon nanodots or gold nanoparticles, for applications in biosensing, payload delivery, or providing a direct therapeutic benefit by binding to their specific targets [21]. One additional disease state where aptamers may offer a unique opportunity is anticoagulation/antiplatelet therapy and monitoring.

## 2. Aptamer Clinical Research in Anticoagulation

### 2.1. Summary of the Coagulation and Clotting Cascades

**Figure 2:**
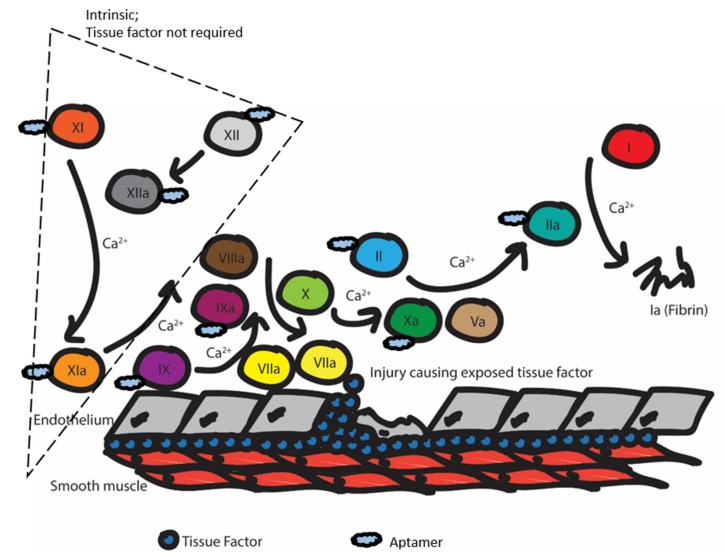
Illustration of the coagulation cascade upon vascular endothelium injury. Aptamers have been isolated to inhibit clotting factors in both the extrinsic and intrinsic pathways of the coagulation cascade. Different inhibition stages ultimately lead to the inhibition of insoluble fibrin (Factor Ia) formation. The blue cloud shapes represent aptamers that have been identified to inhibit specific clotting factors. Each Roman numeral represents the corresponding clotting factor.

In brief, anticoagulation agents work by interrupting the coagulation cascade. This cascade is triggered when the endothelium is damaged, and the tissue factor (TF) is exposed (Figure 2). Then, factor VIIa responds and converts factor IX to IXa and factor X to Xa. Factor IXa complexes with factor VIIIa to further convert factor X to Xa. Factor Xa complexes with factor Va to convert factor II (prothrombin) into factor IIa (thrombin). In turn, thrombin converts factor I (fibrinogen) to factor Ia (fibrin). Calcium is necessary for the activity of many of the above clotting factors. Finally, the fibrin will form a mesh to promote clotting, and fibrin and fibrinogen will interact with platelet receptors to promote adhesion and form the clot. Many current drugs on the market work by inhibiting thrombin or factor X, or inhibit vitamin-K reductase, which is needed to carboxylate several factors [22].

**Figure 3:**
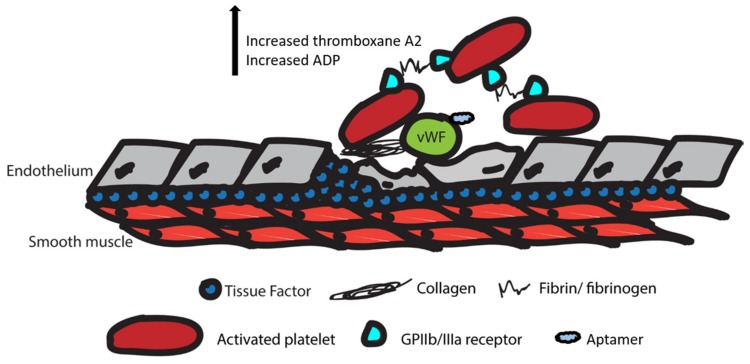
Illustration of the clotting cascade upon vascular endothelium injury. VWF: Von Willebrand factor; ADP: adenosine diphosphate. VWF and collagen activate platelets. Upregulated GPIIb/IIIa receptors on activated platelets increase the release of thromboxane A2 and ADP, and additional platelets are activated to promote clot formation at the site of injury. The blue cloud shape represents aptamers that have been identified to inhibit the Von Willebrand factor and stops platelet activation.

In addition to anticoagulation, some drugs act on the clotting cascade. The clotting cascade is closely related to the coagulation cascade (Figure 3). In the coagulation cascade, platelets are activated by the Von Willebrand factor (VWF) and collagen fibers exposed by initial endothelial injury. The activated platelet then upregulates GPIIb/IIIa receptors and releases thromboxane A2 (TxA2) and ADP to promote activation of additional platelets. Fibrin produced by the anticoagulation cascade and fibrinogen bind to GPIIb/IIIa receptors on platelets to promote adhesion to other platelets. Ultimately, this leads to the aggregation of platelets and clot formation at the site of injury [22].

Since there are a relatively large amount of proteins that are either directly or indirectly involved in the coagulation and clotting cascades, many of them have been investigated as therapeutic targets for inhibition by nucleic acid aptamers. The following sub-section summarizes different anticoagulation aptamers that had progressed into various stages of the pre-clinical testing and clinical trial (Table 1).

### 2.2. Anticoagulation Aptamers

#### 2.2.1. Von Willebrand Factor Inhibitors

ARC-1779 is a DNA aptamer developed by the Archemix Corporation to target the human von Willebrand factor (vWF) [23]. vWF interacts with GP1b on platelets to affect the P2Y12 receptor, which leads to platelet activation. By inhibiting the von Willebrand factor, ARC-1779 blocks platelet activation and platelet aggregation, as well as thrombin generation. ARC-1779 is a 40-nucleotide aptamer that underwent PEGylation and methylation to increase the duration of action in vivo [23]. The clinical utility of ARC-1779 was investigated for cerebral embolism and thrombotic thrombocytopenic purpura (TTP). The high dose completely blocked the target domain on the vWF, whereas the low dose (0.5 µg/ mL) did not, suggesting that the relationship between ARC-1779 and vWF inhabitation may be dose-dependent. Also, ARC-1779 did not resolve all the features of TTP during the trials [41]. During this study, the aptamer did not have any significant bleeding events, and was generally well tolerated. In 2009, phase II studies for clinical use in TPP was completed. However, the sponsor decided to withdraw the study for further investigation.

Matsunaga et al. developed several DNA aptamers that bind to the von Willebrand factor. In order to improve the variety of the genetic library, an unnatural nucleotide, 7-(2-thienyl)imidazo[4,5-b]pyridine (Ds), and its partner, 2-nitro-4-propynylpyrrole (Px), were introduced into the library [18]. Three DNA aptamers—Rn-DsDsDs-44, ARC1172-41, and Pr-DsDsDs-40—were characterized and then modified. Rn-DsDSDs-44 had the highest affinity for vWF (K_D_ = 75 pM), followed by ARC1172-41, and then Pr-DSDSDS-40. The Rn-DsDsDs-44 vWF complex had the highest stability at physiologic temperature. ARC1172 and Rn-DsDsDs-44 both underwent mini-hairpin modification to produce ARC1172-50mh2 and Rn-DsDs-51mh2, respectively to improve the stability of each aptamer. Both of the modified aptamers had greater percentages remaining after a 72-hour incubation in human serum at physiologic temperature compared to the parent aptamers, thus confirming its enhanced nuclease resistance [18].

Nimjee et al. recently developed a new anti-VWF RNA aptamer named DTRI-031 [24]. This aptamer was truncated to a 30-mer from the original 60-mer candidate aptamer. A 5-uracil tail was added to the 3’ position for antidote oligonucleotide binding. DTRI-031 was able to inhibit platelet aggregation in whole blood and prevent thrombosis in mice models in a dose-dependent way. Additionally, DTRI-03 was able to induce vascular recanalization in mouse and dog carotid arteries. The antidote oligonucleotide was also able to reverse the antiplatelet and anticoagulation effects of DTRI-031 [24].

#### 2.2.2. Factor II/ IIa Inhibitors

ARC2172, also known as NU172, is a DNA aptamer that is produced by ARCA Biopharma and Nuvelo [25]. This aptamer was designed as a direct thrombin (factor II) inhibitor. Thrombin is the activated form of prothrombin and is responsible for converting fibrinogen into fibrin. By decreasing the production of fibrin, the fibrin mesh does not form, which allows platelets to adhere to start clot formation. NU172 is a 26-nucleotide unmodified DNA aptamer [26]. Due to the unmodified nature of NU172, it has a relatively short duration of action, with a half-life of 10 min in vivo [22]. The reported short half-life was consistent with the literature [17]. The anticoagulation effect of this aptamer was determined by the activated clotting time (ACT). The dose-dependent anticoagulating effect of this aptamer was seen during Phase 1b trials [25]. No adverse events were reported in either of the phase I trials. An open-label small-size phase II study was started, but the current status of the study is unknown (U.S National Library of Medicine, Clinical trials.gov Identifier: NCT00808964). 

Muller et al. investigated the two DNA aptamers (HD1 and HD22) that bind to the exosite 1 and exosite 2 domain of thrombin, respectively [27]. In combination, the bivalent aptamer (HD1-22) binds to thrombin with a very high affinity of Kd = 0.65 nM. They were also able to confirm the bivalent aptamer’s anticoagulant activity that is close to bivalirudin and superior to argatroban. An antidote-oligodeoxynucleotides was also developed to reverse HD1-22 anticoagulation effect in in vitro testing [27].

Bompiani et al. developed an optimized RNA aptamer, termed R9d14t, which can bind to both prothrombin (factor II) and thrombin (factor IIa) with high affinity (10 nM and 1 nM, respectively) [31]. It inhibited both thrombin formation and coagulation activities mediated by thrombin exosite I. The research group also developed a complementary oligonucleotide antidote to reverse the anticoagulation activities of R9d14t in in vitro settings [31]. No clinical studies have been conducted on R9d14t at this point.

Kotkowiak et al. recently modified a previously identified thrombin binding aptamer RE31 with unlocked nucleic acid (UNA), locked nucleic acid (LNA), and β-L-RNA [28,42]. Modifications with UNA at position 15 T residual and LNAs altered the melting temperature of RE31 and generated twofold higher anticoagulation activity. All of the tested bases modifications did not change the G-quadruplex structure, and also increased the aptamer stability in human serum [28].

Wakui et al. reported using a variant of CE SELEX—microbead-assisted capillary electrophoresis (MACE) SELEX—to identify several thrombin-binding DNA aptamers with nanomolar affinity [29]. The reported aptamer, M08, also demonstrated 10 to 20-fold higher anticoagulation activity than other earlier thrombin binding aptamers in in vitro studies. The author also developed an antidote system for the M08 aptamer [29].

Most recently, Zhou et al. reported using a predetermined DNA nanoscaffold to identify a bivalent DNA aptamer (145-mer) that can bind to both the exosite I and exosite II of thrombin with femtomolar affinity [30]. The bivalent aptamer was able to produce an anticoagulation effect at as low as 5-nM aptamer concentration in human plasma samples.

In addition to identifying novel thrombin-binding aptamers, several research groups recently reported using novel strategies to investigate the anticoagulation activities of HD1 and HD22 under various conditions. Derszniak et al. compared the effects of HD1 and HD22 under flow conditions. The author concluded that HD1 demonstrated stronger anti-thrombotic agents, while HD22 demonstrated weaker antiplatelet and anticoagulation activities due to its binding to exosite II [43]. Lin et al. utilized graphene oxide (GO) to adsorb HD1 and HD22 aptamers via π–π stacking [44]. The GO–aptamer assembly was tested to show superior anticoagulation activity to that of traditional anticoagulant drugs in vitro and in the animal model. The author also reported the high biocompatibility of the assembly in in vitro cytotoxicity and hemolysis assays [44]. Krissanaprasit et al. reported using an in silico design to construct RNA origami containing multiple thrombin-binding RNA aptamers [45]. After introducing the 2’-fluoro modification in C and U nucleotides, the RNA origami structure showed increased resistance to nuclease degradation and also greater anticoagulant activity than free aptamers, which can also be reversed by using ssDNA antidotes [45]. Amato et al. investigated the effect of the different linkers in constructing a dimeric HD1 aptamer [46]. Adenosine or thymidine residues and glycerol moiety were used as the linker in joining the two aptamers. The authors reported the linkers, although they did not alter the global conformation of the aptamers; the binding affinities were different among linker constructs. The authors concluded that the highest affinity dimeric construct demonstrated the highest anticoagulant activity and the most resistance to degradation in biological samples [46].

#### 2.2.3. Factor IXa Inhibitors

REG1, also known as pegnivacogin, is an RNA aptamer that acts as a direct factor IXa inhibitor [32]. A factor IXa inhibitor will prevent the formation of the IXa/VIIa complex, which is an activator of factor Xa. Factor Xa is the rate-limiting enzyme for coagulation, and by inhibiting the VIIa/IXa complex, the process is considerably slower. Factor IXa inhibitors have been investigated for their efficacy within anticoagulation and the simultaneous decreased risk of bleeding. Pegnivacogin is a PEGylated RNA aptamer composed of 31 nucleotides [32]. After administration, pegnivacogin reaches a maximum concentration in the plasma within 5 min or less, and can last for over 24 hours at doses of 0.7 mg/kg. An activated partial thromboplastin time (aPTT) is used to determine the efficacy of pegnivacogin. At doses of 0.7 mg/kg or greater, the aptamer resulted in a 2.5-times increase in aPTT from baseline. In addition to pegnivacogin, its reversal, anivamersen, was developed. Anivamersen is a 15-nucleotide RNA sequence, and it binds to its target using Watson–Crick base pairing [32]. The relationship between anivamersen and the reversal of pegnivacogin is dose-dependent, and the anticoagulation effect is reversed within 5 min of administration of anivamersen in a 1:1 ratio. Pegnivacogin has already undergone phase Ia, Ib, Ic, IIa, and IIb clinical trials. The phase IIb clinical trial studied patients undergoing PCIs or CABGS, and was deemed the RADAR trial. However, enrollment was stopped early after three patients had a severe allergic reaction in the REG1 arm [34]. The investigator later concluded the allergic reaction was due to patients’ pre-existing antiPEG antibodies, and was not due to the aptamer itself [47,48]. The RADAR trial determined that pegnivacogin, in combination with its reversal agent, may lower the risk of bleeds in comparison to unfractionated heparin during artery sheath removal.

Additionally, a phase 3 trial comparing the efficacy of REG1 to bivalirudin in preventing periprocedural ischemic was terminated due to formulation-induced allergic reactions and no evidence of efficacy [33]. During the REG 1 studying phases, the subcutaneous delivery of pegnivacogin in combination with the intravenous delivery of its reversing agent (REG2) was also studied in healthy individuals and showed no toxicities [35]. More recently, an in vitro study was published that showed a reduction in platelet aggregation as well as reversal when anivamersen was introduced [49]. A follow-up post-clinical trial study showed that antiPEG antibodies interacted with the PEGylated RNA aptamer and directly inhibited the anticoagulation properties of the aptamer both in vivo and in vitro [50].

#### 2.2.4. Factor Xa Inhibitors

The RNA aptamer 11F7t was first reported in 2010 as a factor Xa inhibitor [36]. It binds to factor Xa with high affinity (K_D_ = 1.1 nM) and blocks Xa/ Va assembly. More recently, Gunaratne et al. invested the anticoagulation ability of 11F7t in combination with other factor Xa inhibitors currently on the market, i.e., rivaroxaban (oral), apixaban (oral), edoxaban (oral), or fondaparinux (subcutaneous) in in vitro assays [51]. The combination of 11F7t and Xa inhibitors achieved anticoagulation potency that is equal to unfractionated heparin, which is not achievable by any agents alone. Also, the administration of an inactive decoy Xa protein was able to neutralize the anticoagulation effects. 11F7f and in combination were also not affected by the immunoglobulins that resulted from heparin-induced thrombocytopenia. All of the findings suggested the potential for 11F7f in conjunction with other Xa to be an alternative for unfractionated heparin [51].

#### 2.2.5. Factor XIa Inhibitors

Factor Eleven Inhibitory Aptamer, FELIAP, is a DNA aptamer that was developed by the Canadian Blood Services at the Thrombosis and Atherosclerosis Research Institute, and McMaster University [37]. FELIAP acts as a factor XIa inhibitor. Factor XIa helps to convert factor IX to IXa, leading to preventing the activation of factor X, which will prevent the activation of thrombin. This ssDNA aptamer is 74 nucleotides in length (K_D_ = 1.8 nM) [37]. It was hypothesized that FELIAP binds near the active site of factor Xia, since it competitively inhibited a substrate that was cleaved by factor XIa [37]. This theory was further supported because FELIAP did not prevent the activation of factor XI and inhibited two reactions mediated by factor XIa in in vitro experiments. A thrombin generation assay showed that FELIAP reduced the endogenous thrombin potential, while it increased the meantime to peak of thrombin [37]. No in vivo tests have been performed on FELIAP at this point.

Additionally, Woodruff et al. identified two additional aptamers, 11.16 and 12.7, that noncompetitively inhibit factor XIa. 11.16 is an RNA aptamer with a 29-nucleotide random region sequence, and 12.7 is an RNA aptamer with a 49-nucleotide random region sequence [38]. Aptamer 12.7 inhibits FXI and FXIa, whereas 11.16 was found to inhibit FXIa. Both aptamers significantly inhibited the factor FXIa-mediated activation of factor IX. It was determined that the aptamer bound to an anion binding and serpin bindings sites on the factor XIa catalytic domain. However, the control aptamers that were used showed a small amount of inhibition. The author suspected that the negative charge of aptamers might contribute to the inhibition of this target. Aptamer 12.7 increased the aPTT time by approximately 15 seconds as compared to the control library during an aPTT assay. Aptamer 11.16 showed a similar increase in aPTT time compared to the control library, indicating it may interact with other components within the test [32]. At this point, no further analysis of either aptamer has been conducted.

#### 2.2.6. Factor XII/ XIIa Inhibitors

Woodruff et al. reported an RNA aptamer, R4cXII-1 aptamer (K_D_ = 8.9 nM/0.5 nM), that can inhibit the autoactivation of factor XIIa and the factor XIIa-mediated activation of factor XI [39]. It prolonged fibrin formation and thrombin generation in coagulation assays. However, this aptamer was not able to stop the activation of the plasma kallikrein through factor XII-mediated action [39]. No clinical testing data is available at this time.

#### 2.2.7. Kallikrein Inhibitors

Kall1-T4 is an RNA aptamer developed by the Duke Medical Center [40]. Kall1-T4 targets both kallikrein (K_D_ = 0.88 nM) and prekallikrein (K_D_ = 0.28 nM). Kallikrein is responsible for factor VIIa amplification, which is one of the first components of the coagulation cascade. Prekallikrein is the precursor to kallikrein, and is cleaved by the factor VIIa and kininogen complex. Kallikrein plays a role in the inflammatory response. Kallikrein helps to activate bradykinin, which is responsible for vasodilation and inflammation. This RNA aptamer is 54 nucleotides in length [40]. An activated partial thromboplastin time (APTT) was performed to determine the anticoagulation effects of this aptamer. The results showed a dose-dependent increase in clotting time. During a kallikrein-mediated bradykinin assay, Kall1-T4 was determined to reduce the release of bradykinin significantly. No in vivo trials have been performed with Kall1-T4 thus far.

## 3. Aptamer in Anticoagulation Monitoring

**Figure 4:**
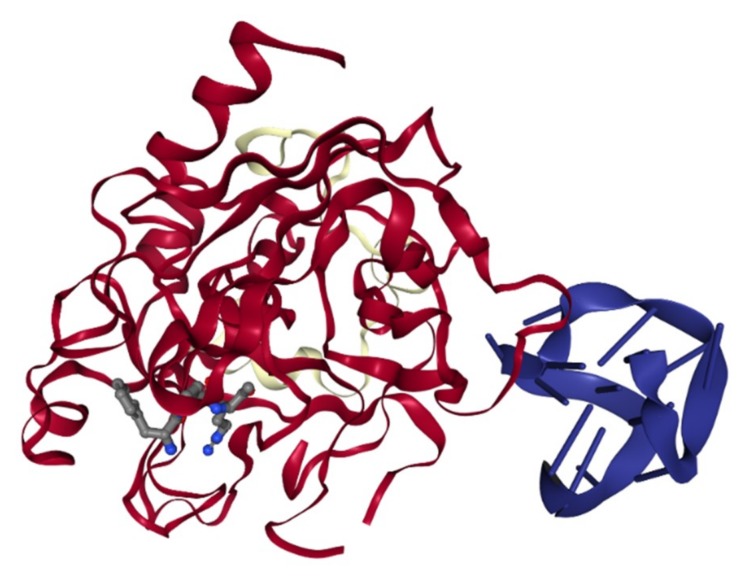
The structure of thrombin interaction with the 15-mer DNA aptamer (PDB 1HUT or NDB PDE013) [53].

The role of aptamer in anticoagulation monitoring has been investigated heavily in the past two decades. The biosensing application of aptamers to detect thrombin has been extensively explored since the identification of the first thrombin-binding DNA aptamer (TBA) in 1992 [52]. One of the most intriguing features of the TBA was the antiparallel G-quadruplex structure formation upon binding to thrombin (Figure 4) [53]. Although other aptamers have been shown to have a G-quadruplex structure, only TBA has the induced G-quadruplex formation upon target recognition [54,55]. It was reported that the G-quadruplex structure in TBA was essential in targeting recognition and its inhibitory effect [53,56]. The uniqueness of the TBA leads to extensive research on its utility in biosensors utilizing various optical, electrical, electrochemical, and mass transferred principles. This topic was reviewed by Deng et al. and Ong et al. recently [57,58].

In contrast to the original research of NU172, HD1, and HD2 as previously discussed therapeutic agents, Trapaidze et al. interrogated their selectivity using surface plasmon resonance measurement [26]. HD22 fit a 1:1 aptamer to thrombin interaction, whereas HD1 and NU172 interacted with thrombin at multiple sites and fit a heterogeneous analyte binding model. It was reported that each aptamer could detect thrombin in the nanomolar range. Individually tested aptamers were introduced to albumin, and both HD1 and NU172 produced a signal in the presence of albumin alone. All three aptamers were able to detect thrombin in the presence of albumin during this experiment, thus confirming their selectivity of thrombin over albumin. HD1 and HD22 were added to the diluted mouse plasma to determine each aptamer’s sensing ability in a more complex environment. HD1 provided similar signals on the SPR sensorgram with or without thrombin, but HD22 showed signal peaks corresponding to the concentration of thrombin. The author concluded that HD22 had the highest potential to be explored as a biosensor for thrombin [26].

In addition to the detection of clotting factors for thrombotic events, recently researchers have also begun investigating using aptamers to measure levels of direct oral anticoagulants. Direct oral anticoagulants (DOACs), such as direct thrombin inhibitors (dabigatran) and direct factor Xa inhibitors (e.g., apixaban) were developed as an alternative to the traditional vitamin-K antagonist (warfarin). Most patients do not require therapeutic monitoring for DOACs. However, it can be useful in managing surgical patients and patients with multiple comorbidities [59]. Currently, dabigatran can be monitored with thrombin time (TT) with high sensitivity.

On the other hand, direct factor Xa inhibitors can only be measured with surrogate tests, such as anti-Xa level and thrombin generation assays. These assays are not routinely used due to a lack of extensive clinical studies [59,60]. Traditional liquid chromatography coupled with mass spectroscopy may also be used to precisely measure the drug level in human plasma, although it is labor-intensive, time-consuming, and requires costly equipment [61].

In 2018, Alhojani et al. used the SELEX process to identify four single-stranded DNA aptamers—DGB-1, DBG-2, DBG-4, and DBG-5—that could bind to dabigatran [62]. DBG-1 had the highest binding affinity to dabigatran, followed closely by DGB-5. DBG-1 underwent modification before it was immobilized on a gold-covered electrode that detected current. Dabigatran was added to the probe solution in varying concentrations. The change in target concentration showed a corresponding reduction of the electric current. Additionally, a conformational change was suspected when the dabigatran molecule bound to the aptamer. The author concluded that this aptamer has the potential for therapeutic monitoring of dabigatran as well as being a potential reversal agent with further exploration and research [62].

## 4. Conclusions and Future Perspective

Since the first description of the thrombin-binding DNA aptamer, multiple aptamers have been isolated to target key clotting factors and co-factors for the treatment and monitoring of thrombotic events. [53]. Researchers have utilized various techniques, such as base modification and unnatural bases library expansion to modify the basic building blocks of nucleic acid aptamers, resulting in increased resistance to nuclease degradation and body retention. However, the lack of approved aptamer-based anticoagulation agents suggested that challenges remain in therapeutic uses of aptamers in the field. Likewise, although aptamers can act as capturing elements in different biosensing platforms, the road to fully commercialized aptamer-based rapid drug monitoring technologies is still somewhat distant. One crucial point to be noted is that aptamer screening and application is a highly multidisciplinary field of research. Collaborations between biologists, chemists, and engineers are essential to the success of aptamer-based technologies.

## Figures and Tables

**Table 1:** Summary table of different pre-clinical and clinical stages anti-thrombotic aptamers.

Aptamer	Type	Target	Developer	Evaluation Stages	Reference
ARC-1779	DNA	Von Willebrand Factor	Archemix	Phase II	[23]
Rn-DsDsDs-44	Unnatural DNA	Von Willebrand Factor	TagCyx Biotechnologies, RIKEN Center for Life Science Technologies	Pre-clinical	[18]
DTRI-031	RNA *	Von Willebrand Factor	Duke Medical Center	Pre-clinical	[24]
NU172	DNA	IIa	ARCA Biopharm	Phase II	[25,26]
HD22	DNA	IIa	University of Bonn	Pre-clinical	[27]
HD1-22	DNA	IIa	University of Bonn	Pre-clinical	[27]
RE31	Modified DNA	IIa	Polish Academy of Sciences	Pre-clinical	[28]
M08	DNA	IIa	The University of Tokyo	Pre-clinical	[29]
ThAD	DNA	IIa	Arizona State University	Pre-clinical	[30]
R9d14t	RNA *	II/ IIa	Duke Medical Center	Pre-clinical	[31]
REG1	RNA *	IXa	Regado Biosciences	Phase III	[32,33,34]
REG2	RNA *	IXa	Regado Biosciences	Phase I	[35]
11F7t	RNA *	Xa	Duke Medical Center	Pre-clinical	[36]
FELIAP	DNA	XIa	McMaster University, Canadian Blood Services Thrombosis and Atherosclerosis Research	Pre-clinical	[37]
11.16	RNA *	XIa	Duke Medical Center	Pre-clinical	[38]
12.7	RNA *	XIa	Duke Medical Center	Pre-clinical	[38]
R4cXII-1	RNA *	XII/XIIa	Duke Medica Center	Pre-clinical	[39]
Kall1-T4	RNA *	Kallikrein	Duke Medical Center	Pre-clinical	[40]

* Note: RNA aptamers contain fluoro-modified cytosine and uracil nucleotides. Each Roman numeral represents the corresponding clotting factor.
